# Family Caregivers’ Involvement in Caring with Cancer and their Quality of Life 

**DOI:** 10.31557/APJCP.2019.20.6.1735

**Published:** 2019

**Authors:** Sevcan Toptas Kilic, Fatma Oz

**Affiliations:** 1 *Hacettepe University Faculty of Nursing, Psychiatric Nursing Department, Ankara, *; 2 *Near East University, Faculty of Nursing, Mersin, Turkey. *

**Keywords:** Oncology, caregiver, quality of life

## Abstract

**Background:**

Cancer is a chronic disease and a major health problem. It affects both patients and their family caregivers multidimensionally. The family caregivers may be affected by not only the disease process but also hospital policies, economic difficulties, accessibility and communication of health care service and can be in need of help. This process may affect their quality of life. However, there have not been enough studies on quality of life of family caregivers of patients with cancer in Turkish culture. Therefore, this study aimed to evaluate the quality of life of family caregivers of patients with cancer in Turkey.

**Objectives:**

The purpose of study was to evaluate the quality of life of family caregivers with cancer patients in Turkey.

**Methods:**

Participants consist of the family caregivers who volunteered to take part in this descriptive study from 11 hospitals (n =378) which has a daily chemotherapy units and located within the boundaries of Ankara, Turkey. ‘Sociodemographic Characteristic Form’ and ‘Quality of Life Scale-Family Version were used as data collection tool. The Kruskal-Wallis and Mann-Whitney U, tests were used for data analysis.

**Resultes:**

It is found that there are statistically significant difference among the factors of gender, employment status, income level, and whether caregivers reside with their patients. Family caregivers’ quality of life is negatively affected during the caregiving process (p < 0.05)**. **

**Conclusion:**

The results indicate that family caregivers’ quality of life are negatively affected to care process. The results of this research are important as they highlight the need to also consider family caregivers’ quality of life when caring for patients, and study highlight possible areas in which support can be provided for family caregivers of cancer patients in Turkey.

## Introduction

Cancer is a chronic disease and a major health problem (Tamayo et al., 2010). It continues to be among the most feared life-threatening diseases despite significant advances in diagnosis and treatment with technological advancement (Güllü and Zengin, 2009). It is the second most common disease in many countries, including Turkey, not only threatening death but also affecting the structure and function of families (Uğur and Fadıloğlu, 2012). It affects both patients and their family caregivers multidimensionally. The family caregivers may be affected by not only the disease process but also hospital policies, economic difficulties, and accessibility and communication of health care service and can be in need of help (Given et al., 2012; Ferrell et al., 2013; Chen et al., 2018). Family caregivers (FCs) of patients with cancer may experience a host of problems, such as anger, hopeless, alone, fear, anxiety, burden, and depression (Chang et al.,2013; Stajduhar, 2013; Effendy et al., 2014). Cancer is no longer considered an acute disease as the survival rates have increased due to improvements in cancer diagnosis, treatment, and care. During cancer treatment, family caregivers play an important role (Kim and Given, 2008). Family caregivers are those who provide uncompensated care in the home and who have a pre-existing relationship (either through friendship or kinship) to the person for whom care is being provided. They assist patients in addressing physical, emotional, and medical problems (Northouse et al., 2010; Mosher et al., 2013). This care may be in any of the quality-of-life (QOL) domains including physical, social, psychological, and spiritual care. Like cancer patients, FCs themselves have diverse needs and health concerns. Caregiver needs and access to resources vary on the basis of many factors, including gender, age, culture, education, economics, and geographic setting (Tamayo et al., 2010). Studies indicate that that, when patient distress increases and symptoms can no longer be controlled, family caregivers experience burden, depression, social isolation, difficulty in concentration, and anxious tendencies (Skarstein et al., 2000).

In addition, family caregivers develop physical problems such as indigestion, changes in appetite, irregular eating habits, headaches, chronic fatigue, weight gain or loss, or muscle pain, resulting in poor quality of life (QoL) (Kitrungrote and Cohen, 2006; Çivici et al., 2011; Turkoğlu and Kilic, 2012; Wadhwa et al., 2013;Kim et al., 2015). Research clearly indicates that the problems experienced by patients with cancer also negatively affect their caregivers. This may cause their QoL to decrease (Park et al., 2013; Sun Young et al., 2015). Therefore, efforts should be made to reduce the burden on family caregivers and to decrease their emotional problems to allow them to provide optimal support and effective long-term care (Pinkert et al., 2013)

As a result, studies have documented that family caregivers feel they are not adequately supported by healthcare professionals. Nurses, as health professionals, can have a positive impact on the well-being of caregivers. Oncology nurses are important members of the cancer team and remain key to the success of patients and family caregivers in providing high-quality, person-centered care. Nurses evaluate QoL of family caregivers and value the opportunities to use a standardized, consistent approach that enables caregivers to report distress (Ferrel and Grant, 2005). However, there have not been enough studies on QoL of family caregivers of patients with cancer in Turkish culture. Therefore, this study aimed to evaluate the QoL of family caregivers of patients with cancer in Turkey. After evaluating their QoL, it also aimed to reflect the results in the nursing care of oncology nurses. The following questions guided this study: 

1. What are family caregivers’ quality of life? 

2. Which sociodemographic family caregivers’ descriptive features are associated with these Quality of life? 

3. Do family caregivers’ views on experiences about care with cancer patient? 

## Materials and Methods

*Study Design and Sample*

This study was conducted using a descriptive method. Units daily patients list were skimmed through for eligibility patients who came with a caregivers were approached. The caregivers of this patients were invited to participate in study if they were aging 18 years and older, literate, and being primary caregiver of the cancer patient. The participants were family caregivers who accompanied patients with cancer when the latter received daytime treatment at 11 chemotherapy units in Ankara. The required sample size was calculated using the “finite population sampling” formula, and the total number was calculated to be 378 family caregivers. The study sample consisted of family caregivers who volunteered to participate in our study. The response rate could not be calculated because the sample size was calculated in advance. The study was performed in other clinics until the target number of sample was reached. Participants were interviewed face to face with caregivers in the waiting room. The duration of data collection was approximately 30 min per individual. 

*Data Collection*

The data were collected through face-to-face interviews by researchers in hospital daily chemotherapy units. Researchers were available at the relevant unit to answer possible questions during this period. 

*The sociodemographic characteristic*

The family caregiver sociodemographic characteristic includes age, gender, marital status, education level, work status, and illness treatment characteristics (e.g., diagnosis, duration of caregiving, relationship with patient, type of cancer, chemotherapy cycles, and information about caregivers). There is also 1 open-ended question whether they have experienced affected by care process. 

Quality of Life Scale-Family Version (QoL-FV). The scale developed by Ferrell and Grant. (Ferrel and Grant, 2005). In this study the Turkish version was used. The test–retest reliability coefficient was found (r = .86), and Cronbach’s alpha coefficient for internal consistency was = .90. The QoL-FV is composed of 4 subscales: Psychological and Spiritual Health Status (1, 2, 3, 4, 5, 6, 7, 8, 9, 10, 11), Physical Health Status (12, 13, 14, 15, 16, 17, 18, 19, 20), Approach to Diagnosis Status (21, 22, 23, 24, 25, 26, 27), and Support and Economic Response Status (28, 29, 30, 31). Some questions (1,12–15, 17–28, 30, 31) were reversely encoded. The scale is expressed by collecting the response scores regarding the items of relevant size of total and subscale mean scores and dividing the number of items/questions. The scale is interpreted through total and subscale scores, and a high score indicates high QoL (Okçin and Karadakovan, 2012).

*Ethical Approval*

After written permission was received from the “The University Non-Interventional Clinical Research Ethics” (LUT 12/48 reference number) and hospitals, family caregivers were informed about the study. After receiving written consent from the volunteering family caregivers, questionnaires were conducted until the target sample was reached. 

*Analysis*

The data were analyzed using the SPSS 20.0. Frequencies and percentages were used to determine family caregivers’ descriptive characteristics. The sampling did not show a normal distribution. Therefore, non-parametric tests were used for analysis. Distinctive differences in QoL were assessed using the Mann–Whitney U test for comparisons with two categories, and Kruskal–Wallis one-way analysis of variance was used for comparisons of more than two categories. The Bonferroni correction was used to determine the origin of the differences. Differences between groups were considered significant at a level of p < 0.05.

## Results

*Caregiver Characteristics*

A description of the family caregivers is shown in Table 1. A total of 378 family caregivers were recruited and interviewed. In this study, caregivers were predominantly female (64.3%, n = 243); 75.4% were married. The majority of participants ranged in age from 31 to 50 years (50.8%, n = 192); the majority were primary (29.6%, n = 112) and secondary school (33.6%, n = 127) graduates, 48.4% were unemployed, and the income level of 34.7% of the participants could be classified as low. Regarding the kinship of the family caregivers, one-third (29.1%, n = 110) were participants’ spouses. More than half (53.7%, n = 203) had been caring for the patient for 1–6 hours, one-third (29.4%, n = 111) all day, and more than half (57.4%, n = 217) shared a residence with patients and did not get any support from others during caregiving (48.4%, n = 183). 

*Responses from Family Caregivers Affected by the Caregiving Process*

Regarding the family caregivers’ responses to disease-related processes, 81% were negatively affected, and only 11.9% were not affected. When examining how caregivers’ families were affected by the caregiving process, more than half (55.1%) were affected negatively, and 24.2% were affected positively. While the working lives of almost half of the caregivers (48.1%) were adversely affected, 40.2% of the caregivers reported not being affected. Some of the caregivers explained that the lack of effect was because they had their own businesses or were retired. Most caregivers (63.2%) reported that their social lives were negatively affected whereas 29.6% said that they were not affected (shown in Table 2).

*QOL-FV Family Caregivers (N = 378)*

The family caregivers’ median scores on the five domains are shown in Table 3. The caregivers’ median total QOL-FV score was 4.83 (max = 8.71, min = 0.87). The median subscale scores were as follows: 5.45 (max = 10.0, min = 0.00) for Psychological and Spiritual Health, 5.44 (max = 10.0, min = 0.22) for Physical Health, 2.71 (max = 9:43, min = 0.00) for Approach to Diagnosis, and 4.75 (max = 10.0, min = 0.00) for Support and Economic Response. A comparison of the min–max scores suggested that the Psychological and Spiritual Health subscale was moderate and that the Approach to Diagnosis, Support and Economic Response, and total QoL scores were below average.

As shown in Table 4 there were statistically significant differences between some mean participants scores on account of some variables in the samples. There were no signiﬁcant differences between caregivers’ ages, marital statuses, patients’ cancer types, and QoL scores. There were statistically significant differences in terms of caregivers’ gender, education status, employment status, financial status, relationship with patient, and whether caregivers resided with their patients (p = 0.000, p < 0.05) (Table 4). Female caregivers were found to have lower quality of life than male caregivers. The quality of life scores of the students with primary school education level were lower than those with university education level in “Psychological and Spiritual Health” in subscale. The quality of life scores of “Psychological and Spiritual Health” and “Physical Health” were found to be lower compared to others.

**Table 1:** Descriptive Characteristics (N=378).

Variables	Frequency (n)	%
Gender		
Female	243	64.3
Male	135	35.7
Age		
20-30	69	18.3
31-50	192	50.8
51+	117	31
Marital status		
Single	93	24.6
Married	285	75.4
Education status		
Primary school	112	29.6
Secondary school	127	33.6
High school	89	23.5
Üniversity	43	11.4
Graduate	7	1.9
Employment status		
Employed	145	38.4
Unemployed	183	48.4
Retired	50	13.2
Income status		
Least income expense	131	34.7
Equal to the revenue expenditure	193	51.1
Revenue over expenses	54	14.3
Children		
With children	276	73.2
Without children	101	26.8
Patient Relationship		
Spouse	110	29.1
Mother	92	24.3
Father	58	15.3
Brother/sister	46	12.2
Son/daughter	7	1.9
Others	65	17.2
Duration of caregiving (hour/day)		
1-6	203	53.7
7-12	57	15.1
13-18	7	1.9
19-24	111	29.4
Care support from other family members		
Present	194	51.3
Absent	183	48.4
Living in the same household status		
Yes	217	57.4
No	161	42.6

**Table 2:** Caregivers are Affected by the Disease Process

Affected Situation	Frequency (n)	Percent %
The caregiver’s own life being affected by the disease process		
Not affected	45	11.90
Negative affected	306	81
Affected	27	7.10
The caregiver's family life		
Not affected	57	15.10
Positive affected	84	24.20
Negative affected	208	55.1
No answer	29	7.70
The caregiver’s job life		
Not affected	152	40.20
Negative affected	182	48.10
No answer	44	11.20
The caregiver’s social life		
Not affected	132	29.60
Negative affected	219	63.20
No answer	27	7.20
Total	378	100.00

**Table 3:** Family Caregivers “QoL-Family Version” Mean Scores in Four Domains

Quality Of Life Scale-Family Version	Median (min-max)
Psychological and Spiritüel Well Being	5.45 (0-10)
Physical Well Being	5.44 (0.22-10)
Diagnostic Approach to Status	2.71 (0-9.43)
Support and Economic Impact Status	4.75 (0-10)
Total	4.83 (0.87-8.71)

**Table 4:** Comparison of Mean Scores of the Four Domains Related to Description of Family Caregivers (N=378).

Descriptives		Quality Of Life Scale-Family Version				
		Total Scale Score	Psychological and Spiritüel Well Being	Physical Well Being	Diagnostic Approach to Status	Support and Economic Impact Status
		Median (min-max)	Median (min-max)	Median (min-max)	Median (min-max)	Median (min-max)
Gender	Female (n=243)	4.55 (0.87- 8.71)	5 (0- 10)	5 (0.22- 10)	2.43 (0- 9.43)	4.75 (0- 10)
	Male (n=135)	5.19 (2- 8.58)	5.82 (1.55- 9.82)	6 (1.44- 10)	3.29 (0- 9)	4.75 (0- 9.5)
	Statistical analysis*	U=20.122 p=0.000	U= 20.340 p=0.000	U= 19.061 p=0.009	U=20.527 p=0.000	U=16.086 p=0.756
*Mann Whitney U Test is used						
*Kruskal-Wallis Varyans Analyz is used..						
Education Status	Primary school ^a^(n=112)	4.71 (1.13-8.68)	4.91 (0.82 – 9.55)	5.44 (0.67-9.89)	2.86 (0-9)	4.13 (0- 10)
	Secondary school ^b^(n=127)	4.74 (0.87-8.19)	5.36 (1.64- 8.45)	4.67 (0.22- 9.44)	3 (0-7.86)	4.25 (0- 9.25)
	High school ^c^(n=89)	4.84 (1.19-8.71)	5.27 (0- 9.64)	5 (1.22- 10)	2.71 (0-9.43)	4.75 (0- 8.75)
	Üniversity ^d^(n=43)	4.97 (1.13-8.71)	5.73 (0.82- 10)	5.78 (1.33- 10)	2.43 (0-8)	5.25 (0.5 -9.25)
	Graduate ^e^(n=7)	5.42 (4.06 – 7.45)	5.45 (4.82 – 8.91)	6 (3.22-8.89)	2.86 (1.86-6.14)	5.50 (3.5 -9.25)
	Statistical analysis*	KW= 7.424 p=0.115	KW= 17.043 p=0.002	KW= 8.342 p=0.080	KW= 4.811 p=0.307	KW= 8.906 p=0.063
*Kruskal-Wallis Varyans Analyz is used. Psychological and Spiritüel Well Being: a-d						
Employment status	Employed ^a^(n=145)	4.97 (0.87- 8.58)	5.55 (082- 9.64)	5.89 (0.22-10)	2.71 (0-9)	4.75 (0-9.25)
	Unemployed ^b^(n=185)	4.52 (1.13- 8.71)	4.91 (0-10)	5 (0.67-9.89)	2.57 (0-9)	4.50 (0-10)
	Retiredc (n=50)	5.26 (2- 8.71)	6.18 (1.55- 9.91)	5.50 (1.67-10)	3.36 (0-9.43)	5.75 (0-9.25)
	Statistical analysis*	KW=10.375 p=0.006	KW=15.919 p=0.000	KW=7.914 p=0.019	KW=2.848 p=0.241	KW=5.832 p=0.054
*Kruskal-Wallis Varyans Analyz is used. Total scale: b-a, b-c Psychological and Spiritüel Well Being: b-a, b-c, Physical Well Being :b-a						
Income status	least income expense ^a^(n=131)	4.19 (0.87-8.71)	4.91 (0.82- 9.45)	5 (0.22- 9.44)	2.71 (0- 9.43)	3.75 (0- 9.5)
	equal to the revenue expenditure ^b^(n=193)	4.90 (1.13- 8.58)	5.55 (0- 9.91)	5.56 (0.67-10)	2.57 (0- 9)	5 (0- 10)
	Revenue over expenses ^c^(n=54)	5.56 (1.13- 8.71)	6.50 (0.82-10)	6.94 (1.33- 9.89)	3.43 (0-8.43)	5.50 (1.75- 8.50)
	Statistical analysis*	KW=22.244 p=0.000	KW=25.719 p=0.000	KW=15.998 p=0.000	KW=5.058 p=0.080	KW=25.895 p=0.000
*Kruskal-Wallis Varyans Analyz is used. Total scale: a-b, a-c, b-c, Psychological and Spiritüel Well Being: a-b, a-c, b-c Physical Well Being : c-a, c-b, Support and Economic Impact Status : a-b, a-c						
duration of caregiving (hour/day)	1-6 ^a^(n=203)	5.35 (0.87- 8.71)	5.73 (0.73-10)	6.56 (0.22-10)	3 (0-9)	5.25 (0-10)
	7-12 ^b^(n=57)	4.10 (1.65- 8.71)	5 (0.82- 9.45)	4.78 (0.89-8.89)	1.71 (0-9.43)	4 (0.5- 8.5)
	13-18 s^c^(n=7)	4.94 (2.35- 5.06)	4.91 (3.73- 7.73)	5.44 (1.33-7.89)	1.86 (0.57-4.86)	4 (2.5-4.75)
	19-24 ^d^(n=111)	4.03 (1.13- 8.68)	4.91 (0- 9.82)	4.33 (0.67-9.89)	2.71 (8.57)	4 (0-9.5)
	Statistical analysis*	KW=35.607 p=0.000	KW=17.915 p=0.000	KW=51.939 p=0.000	KW=8.806 p=0.032	KW=24.129 p=0.000
Kruskal-Wallis Varyans Analyz is used. Total scale: a-b, a-d, Psychological and Spiritüel Well Being: a-d, Physical Well Being:a-b, a-d, Diagnostic Approach to Status: a-b, Support and Economic Impact Status: a-b, a-d						
Relationship to patient	Spousea (n=110)	4.18 (113-8.68)	5 (0.82- 9.82)	4.50 (0.67- 9.89)	2.79 (0- 8.57)	4.13 (0- 9.25)
	Motherb (n=92)	4.65 (0.87- 8.58)	5.18 (0- 9.55)	5.44 (0.22-10)	2.14 (0- 9)	4.50 (0-9)
	Fatherc (n=58)	4.87 (2.35- 7.97)	5.41 (1.45- 9.64)	5.56 (1.33-9)	2.71 (0- 6.86)	4.25 (0.5- 9.5)
	Brother/sisterd (n=46)	5.02 (1.68- 8.71)	5.77 (1.64- 9.91)	6 (0.89- 10)	2.93 (0- 9.43)	5.50 (1.25-10)
	Daughter/sone (n=7)	3.58 (2.35- 6.97)	3.91 (2.91- 6.91)	4.33 (3.67-7)	2.43 (0.14- 6.71)	3.25 (0- 7.5)
	Others(n=65)	5.77 (1.55- 8.71)	6.27 (0.73- 10)	7.11 (2.33- 9.89)	3.29 (0- 7.86)	5.75 (0.25-9.5)
	Statistical analysis*	KW=29.889 p=0.000	KW=19.513 p=0.002	KW=34.147 p=0.000	KW=10.626 p=0.059	KW=26.411 p=0.000

Kruskal-Wallis Varyans Analyz is used. Total scale: a-f, b-f, c-f, Psychological and Spiritüel Well Being: c-f, a-f, Physical Well Being: a-f, b-f, Support and Economic Impact Status: b-f, a-f, c-f, a-d

## Discussion

In Turkey, the family plays an important role in patient care. It is a tradition and considered an obligation to take care of a family member who is sick at home as well as during medical care (Hacioğlu et al., 2010; Sercekus et al., 2014). Firstly, this study revealed that QoL scores were lower among female than among male caregivers. This may occur because of the high responsibility of women in society. The caregiving role is considered multi-dimensional and includes the patient, children, housework, shopping, maintenance of social relationships, and children’s education. Society traditionally regards women as playing a role in all these areas in Turkey. Therefore, female caregivers are expected to take on these roles and are also not used to accepting assistance or sharing responsibility. Eventually, this leads to an increased sense of burden and decreased QoL (Scherbring, 2002; Awadalla et al., 2007; Alptekin, 2010). Secondly, our study revealed that there are statistically significant differences among education levels according to mean QoL-FV score. This could be due to increased financial and social opportunities as well as education levels themselves. Caregivers with higher educational attainment may have more knowledge and communication skills, may know more efficient stress management techniques, and may find it easier to cope with problems. Studies suggest that caregivers with lower educational levels are less efficient at meeting patients’ treatment needs, and this could adversely affect their QoL (Gözüm and Akçay, 2005; Gaugler et al., 2005). Thirdly, we found that the QoL-FV scores were lower for unemployed caregivers than for employed or retired caregivers. When not working, caregivers cannot provide economically efficient care, and the lack of gainful employment can lead to a sense of worthlessness. Thus, poorer QoL might be expected among unemployed caregivers, even though this reduces the overall level of responsibility, enabling these caregivers to devote more time to patients. Gaugler et al., (2005) reported different findings from ours that employed caregivers reported feelings of inadequacy with regard to care provision due to their work obligations. This, in turn, increased depressive symptoms and reduced their QoL. Our findings are supported by several studies showing that caregivers with a monthly income less than their expenses obtained the least QoL (Yun et al., 2005; Longo et al., 2006) Next, our study revealed that the QoL of caregivers of patients who had been diagnosed a year or less earlier was lower compared to those who had been diagnosed more than five years earlier. Similarly, in the literature, some studies showed poor QoL among spouses caring for newly diagnosed patients (Neyt and Albrecht, 2006; Görgülü and Akdemir, 2010). Some studies have also indicated an increase in the QoL of both the patient and the caregiver following successful treatment, also indicating that caregivers maintained this process better and used social support more efficiently (Neyt and Albrecht, 2006; Osse et al., 2006; Yoo et al., 2008). Then, we also found that the mean QoL-FV scores were not statistically significant according to diagnosis. This may suggest that the type of cancer with which a patient is diagnosed was less important than the cancer diagnosis itself. This statement suggests that any type of cancer may result in negative feelings among family caregivers as a result of fear, anxiety, uncertainty, and death. Thus, the psychological QoL may be negatively affected.

Next, in our study, the mean QoL-FV scores for caregivers providing 1–6 hours were higher than for those for caregivers providing for longer periods. Some studies have reported that depression and anxiety levels, as well as the burden of caregiving, were closely associated with the caregiving duration during the week (Song et al., 2011; Ito and Tadaka, 2017). Moreover, the caregiving burden tended to decrease over time, though the perceived burden did not change after six months (Yoo et al., 2008). This suggested that caregivers prioritize their patients and neglect their own needs. 

Then, in our study, a statistically significant difference in the total QoL-FV score was found with regard to employment status. Moreover, caregivers whose work was not affected by caregiving obtained significantly lower QoL scores, compared to those whose work was affected. Employed caregivers stated that they had to leave their jobs, retire, or close their workplaces even when they had not planned to do so (Çivici et al., 2011). Furthermore, Longo et al., (2006) conducted their study among 893 caregivers and reported that caregivers had to sell their assets, take out mortgages, or find additional work to meet their patients’ healthcare needs. In contrast, some caregivers mentioned that the cancer-related processes positively affected their families, bringing them closer together and enabling them to engage with each other. This demonstrates that this process might not have a universally negative effect on families but may even strengthen family bonds. 

Lastly, statistically significant differences were found in total QoL scores based on social life during caregiving. In another study, caregivers did not have the time and energy to meet their own needs, relax, engage in leisure activities, and visit their friends or relatives, and this exacerbated their difficulties (Osse et al., 2006; Uğur and Fadıloğlu, 2012). According to our study, it can be concluded that caregivers experienced a physical burden as a result of the change in roles, not having time to themselves, providing care almost all day, and poor work performance as well as an emotional burden based on their perception that caregiving was a liability. The burdens negatively affected the caregivers’ QoL. A negatively affected QoL was considered a risk to the well-being and physical and mental health of caregivers (Song et al., 2011).

*Application*

The study’s results show that care for patients with cancer by family caregivers can be challenging in multiple ways. In contrast, healthcare professionals must have QOL-FV focus in caring for patients with cancer who have family caregivers. Furthermore, they ought to work in an interdisciplinary fashion and provide both patients and family caregivers with adequate information and tools to handle their situation to promote QOL-FV and well-being. Having a QOL-FV perspective gives a variety of possibilities for health-promoting interventions. Such interventions may be related to practical coping, physical activity, and information and communication as well as social network support. The results also indicate that healthcare professionals and researchers should have a family-oriented focus and develop interventions targeted toward the entire family.

In conclusion, in our study, the QoL of family caregivers was significantly low. It was even lower for female caregivers, caregivers with lower socio-economic status and lower education levels, unemployed caregivers, those caring for recently diagnosed patients, those living with patients, those providing care for longer periods, those with immediate family as patients, those who could not fulfill their family responsibilities due to caregiving, and those without any support from other family caregivers. The findings of this study are particularly relevant for nurses caring for patients with cancer who have family caregivers. The results indicate that focusing on the family caregivers’ QOL-FV may give a better indication of their challenges as well as resources. A QOL-FV perspective may give healthcare professionals an indication of the areas of special importance. On this basis, adequate and tailored interventions to promote QOL-FV and health can be developed.

## Conflicting of Interests

Disclosure.

## Author Contributions

Each author participated in the study design, data collection, data analysis, and manuscript preparation, and there are no conflict of interests.

Study design: S.T.K., F.Ö.

Data collection: S.T.K.

Data analysis: S.T.K 

Study supervision: F.Ö.

Manuscript writing: S.T.K.
